# Correction to “Epidemiological Study of Tibial Plateau Fractures Combined with Intercondylar Eminence Fractures”

**DOI:** 10.1111/os.14082

**Published:** 2024-04-17

Lv, H. , Zhang, Q. , Chen, W. , Song, Z. , Zheng, Z. and Zhang, Y. (2020), Epidemiological Study of Tibial Plateau Fractures Combined with Intercondylar Eminence Fractures. Orthop Surg, 12: 561–569. 10.1111/os.12658 32347009 PMC7189054

The findings presented were not able to introduce a predicted model for the risk of combined injuries based on multiple factor analysis as stated in part three of the introduction. This sentence should have read “to confirm the risk factors of these combined injuries based on multiple factor analysis”.

In the results section, which describes the distribution of patients by age group, there is an error in the sentence “The highest proportion age group of tibial plateau fractures included the ages of 45‐54 years, with more men than women for both age groups.” The sentence should read as follows: “The highest proportion age group of tibial plateau fractures included the ages of 45‐54 years, with more men than women for this age group.”

In the results section which describes the distribution of patients by treatment methods, the median and interquartile ranges have been erroneously transcribed as citations to previous references and should read as follows: “The mean hospitalization time was 16 days (median 13). The hospitalization time of patients with intercondylar eminence fractures was 15 days (median 11) and was significantly shorter than that of patients without inter‐condylar eminence fractures – 17 days (median 13) z = 5.821, p < 0.01).

We apologize for these errors.

